# Supplementary material for: The program efficiency of environmental and social non-governmental organizations: A comparative study
Source: PLoS One. 2024 May 14;19(5):e0302835. doi: 10.1371/journal.pone.0302835 (PMC11093323; doi:10.1371/journal.pone.0302835)
Supplement: Attachment — Submitted filename: Response to Reviewers.docx [file pone.0302835.s001.docx]

Dear Dr. Peng,

Thank you for submitting your manuscript to PLOS ONE. After careful consideration, we feel that it has merit but does not fully meet PLOS ONE’s publication criteria as it currently stands. Therefore, we invite you to submit a revised version of the manuscript that addresses the points raised during the review process.

Dear Journal Editor,

I am grateful for the detailed feedback received on my manuscript and the chance to enhance its quality through revisions. The reviewers' insights are highly valued. Rest assured, I am diligently working on addressing the comments and believe that these improvements will bring the manuscript in line with the publication standards of PLOS ONE.

I anticipate submitting the updated version of the manuscript soon for your further review.

Thank you once again for your assistance and direction.

Best Regards,

Sujie Peng

Revision documentation checklist:

- A rebuttal letter that responds to each point raised by the academic editor and reviewer(s). You should upload this letter as a separate file labeled 'Response to Reviewers'.
- A marked-up copy of your manuscript that highlights changes made to the original version. You should upload this as a separate file labeled 'Revised Manuscript with Track Changes'.
- An unmarked version of your revised paper without tracked changes. You should upload this as a separate file labeled 'Manuscript'.

If you would like to make changes to your financial disclosure, please include your updated statement in your cover letter. Guidelines for resubmitting your figure files are available below the reviewer comments at the end of this letter.

Journal requirements:

When submitting your revision, we need you to address these additional requirements.

1. Please ensure that your manuscript meets PLOS ONE's style requirements, including those for file naming. The PLOS ONE style templates can be found at

https://journals.plos.org/plosone/s/file?id=wjVg/PLOSOne_formatting_sample_main_body.pdf and

https://journals.plos.org/plosone/s/file?id=ba62/PLOSOne_formatting_sample_title_authors_affiliations.pdf

2. Thank you for stating the following financial disclosure:

“This work was supported by the UIC Start-up Research Fund [grant number UICR0700043-23].”

Please state what role the funders took in the study. If the funders had no role, please state: "The funders had no role in study design, data collection and analysis, decision to publish, or preparation of the manuscript."

If this statement is not correct you must amend it as needed.

Please include this amended Role of Funder statement in your cover letter; we will change the online submission form on your behalf.

Reply: Thanks, the statement has been updated by the end of the cover letter, stating that “this work was supported by the UIC Start-up Research Fund. The funders had no role in study design, data collection and analysis, decision to publish, or preparation of the manuscript.”

3. We note that you have indicated that there are restrictions to data sharing for this study. PLOS only allows data to be available upon request if there are legal or ethical restrictions on sharing data publicly. For more information on unacceptable data access restrictions, please see http://journals.plos.org/plosone/s/data-availability#loc-unacceptable-data-access-restrictions.

Before we proceed with your manuscript, please address the following prompts:

a) If there are ethical or legal restrictions on sharing a de-identified data set, please explain them in detail (e.g., data contain potentially identifying or sensitive patient information, data are owned by a third-party organization, etc.) and who has imposed them (e.g., a Research Ethics Committee or Institutional Review Board, etc.). Please also provide contact information for a data access committee, ethics committee, or other institutional body to which data requests may be sent.

b) If there are no restrictions, please upload the minimal anonymized data set necessary to replicate your study findings to a stable, public repository and provide us with the relevant URLs, DOIs, or accession numbers. For a list of recommended repositories, please see

https://journals.plos.org/plosone/s/recommended-repositories. You also have the option of uploading the data as Supporting Information files, but we would recommend depositing data directly to a data repository if possible.

We will update your Data Availability statement on your behalf to reflect the information you provide.

Reply: Besides, the dataset utilized in this research is owned by a third-party entity, the CNRDS database, and legal constraints prevent its sharing. One can gain access to the dataset through the official website of the database (https://www.cnrds.com/Home/Login) or by reaching out to the corresponding author.

4. PLOS requires an ORCID iD for the corresponding author in Editorial Manager on papers submitted after December 6th, 2016. Please ensure that you have an ORCID iD and that it is validated in Editorial Manager. To do this, go to ‘Update my Information’ (in the upper left-hand corner of the main menu), and click on the Fetch/Validate link next to the ORCID field. This will take you to the ORCID site and allow you to create a new iD or authenticate a pre-existing iD in Editorial Manager. Please see the following video for instructions on linking an ORCID iD to your Editorial Manager account: https://www.youtube.com/watch?v=_xcclfuvtxQ

Reply: Thanks for your comment, the ORCID ID has been updated on the EM system.

5. Please review your reference list to ensure that it is complete and correct. If you have cited papers that have been retracted, please include the rationale for doing so in the manuscript text, or remove these references and replace them with relevant current references. Any changes to the reference list should be mentioned in the rebuttal letter that accompanies your revised manuscript. If you need to cite a retracted article, indicate the article’s retracted status in the References list and also include a citation and full reference for the retraction notice.

Reply: Thanks for your suggestion, it can be confirmed that the reference list is complete and correct. No retracted article has been cited.

Reviewers' comments:

5. Review Comments to the Author

Please use the space provided to explain your answers to the questions above. You may also include additional comments for the author, including concerns about dual publication, research ethics, or publication ethics. (Please upload your review as an attachment if it exceeds 20,000 characters)

Reviewer #1:

1. In the hypothesis development part, it needs to be clarify why the research focuses on professionalism to measure efficiency and not the other factors such as the impacts of the organisations in delivering services to the community within limited resources. There are many previous research in other countries and China shows that professionalism, or in development word is called, NGO-ization, leads to inefficient and ineffective in downward accountability. Therefore, it would be good to explore about the contestation of professionalism in the literature review of hypothesis development before come with the results.

Reply: Thank you for your insightful comments. As detailed in Panel D of Table 1, the efficiency assessment is based on the percentage of funds that NGOs devote to the execution of projects central to their mission. The elements shown in Panels A and B represent potential factors impacting NGO efficiency. Additionally, I’ve introduced a new subsection on NGO professionalism within **page 9** of the literature review, prior to the formulation of our hypothesis. Also, part of the hypothesis development has been updated.

Reviewer #2:

2. This study aims to investigate the factors influencing the program efficiency of NGOs in China and compare the differences between environmental and social NGOs. The author has not included findings from relevant and recent studies as this is dependent on the literature primarily before 2015. This is noted throughout the work. This requires updation.

Reply: Thank you for your valuable suggestion. I have now updated the majority of the citations to reflect more recent sources (the sources in or after 2015). Nevertheless, a few studies, such as Kreutzer and Jäger (2011), Luo et al. (2012), and Hwang and Powell (2009), were not updated as these works established robust conceptual bases for specific concepts, were published in high-impact journals, and/or demonstrated substantial citation levels.

3. Author fails to provide a demographics of the voluntary sector in China as many things needs to be understood in the context

Reply: Thank you for your valuable input. I have added a new section to introduce the demographics of the voluntary (nonprofit) sector in China. This section can be found at the beginning of the literature review (page 5).

4. Citation required Pg 11-‘However, various studies have discussed measurements of NGO efficiency’

Reply: I am grateful for your constructive recommendation. In alignment with your advice, I have added the studies by Özbek (2015) and Polonsky et al. (2016) at the end of the sentence on page 2.

5. Why the author wants to compare between environmental and social NGOs? Is there any specific reason which is comparable between? Also, in the results/discussion also this comparison/contrast is not outlined.

Reply: Thanks for your valuable feedback. In response, I have elaborated on the rationale for comparing ENGOs and social NGOs at the conclusion of the introduction (page 4) and at the outset of the discussion (page 26). In particular, comparative research is crucial for understanding the macro-social variations in NGOs, revealing factors behind their global expansion and adaptation. Pioneering work by Lazzarini and Musacchio (2018) and Luong et al. (2019) shows that ownership structures affect organizational performance. However, following the Momin's (2013) classification, there is a research gap at the organizational level in non-western contexts, particularly between ENGOs (focused on sustainability and conservation) and social NGOs (addressing societal issues). Also, this research suggests that the determinants of performance (program efficiency) can differ among nonprofits, dependent on their mission focus. Thus, a comparative analysis of the efficiency of these two types of NGOs in China is necessary.

Additionally, we have enhanced the results and discussion sections by integrating a greater number of comparisons. For instance, in page 29, NGOs addressing social issues are often seen as potential threats by the state, leading to imposed restrictions. The government, despite recognizing the positive contributions of NGOs, has limited their expansion, using legal measures to control them. This has resulted in an inverse relationship between government funding and program efficiency, particularly in NGOs dealing with social issues.

6. It will be good if the author could communicate the components with which he/she measured the efficiency? If there is a Govt rating, will that be the indicator of efficiency? And if yes, are those components taken into consideration for measurement?

Reply: Thank you for your feedback. As shown in Panel D of Table 1, we measure efficiency by the proportion of funds that NGOs allocate towards the execution of mission-related projects. Furthermore, the performance feedback from the government and other components presented in Panels A and B are factors influencing NGO efficiency. They are not indicators of efficiency themselves.

7. Nproper and NStaper you mentioned the mean value is 0.01 which is not clear. What is that mean average? Is it the mean from the sum?

Reply: Thank you for your constructive suggestion. The incorrect average number concerning staff with work experience in governmental agencies has been updated to reflect the correct information as per Table 2. In particular, the findings propose that, within the scrutinized NGOs, a mean of 0.08 and 0.35 of the workforce have a background of either past or current employment with the government, respectively.

8. Unlike social NGOs, the efficiency of environmental NGOs is negatively and significantly associated with the registration level- What does that implies? Is the efficiency low for those organisations at low category of registration (Municipal)?

Reply: Thanks for your suggestion, in the main text, I have now added an explanation after this sentence. Specifically, it implies that: in terms of social NGOs, those possessing elevated registration levels (for instance, those registered at national governmental bodies) demonstrate enhanced efficiency relative to those with diminished registration levels (such as those registered at municipal governmental bodies).

9. Likewise, it was also shown that managers who received higher and more consistent ratings from both themselves and others tended to have more positive outcomes (Ashford et al., 2018; Shull, 2010). The results of our regression statistics is consistent with these notions- This part is not clear. In the definition of variables, you have selected for this study, ‘feedback’ is defined as the variable which represent the NGOs evaluation from National body. Is this consistent with the rating of managers you have mentioned here?

Reply: Thank you for your feedback. I have now incorporated references from organizational studies, specifically Kotiloglu et al. (2021) and Ref and Shapira (2017), to discuss how performance feedback can influence organizational behavior, such as improving NGO efficiency (page 27).
